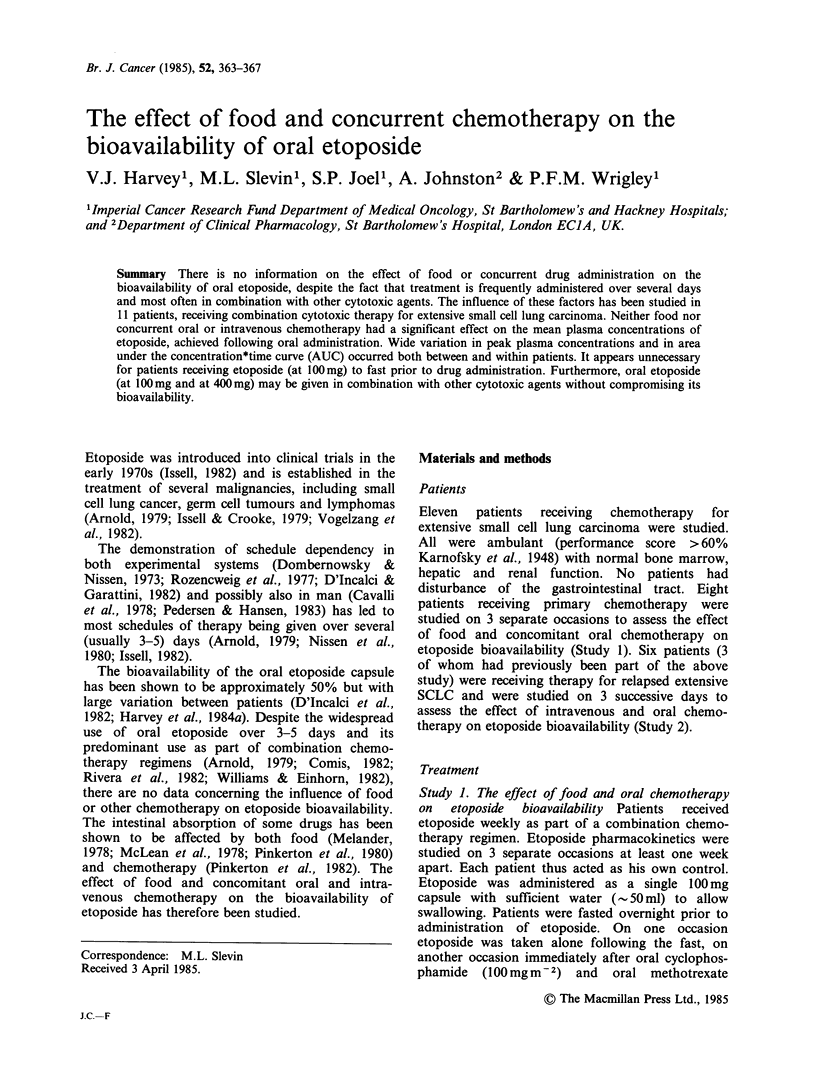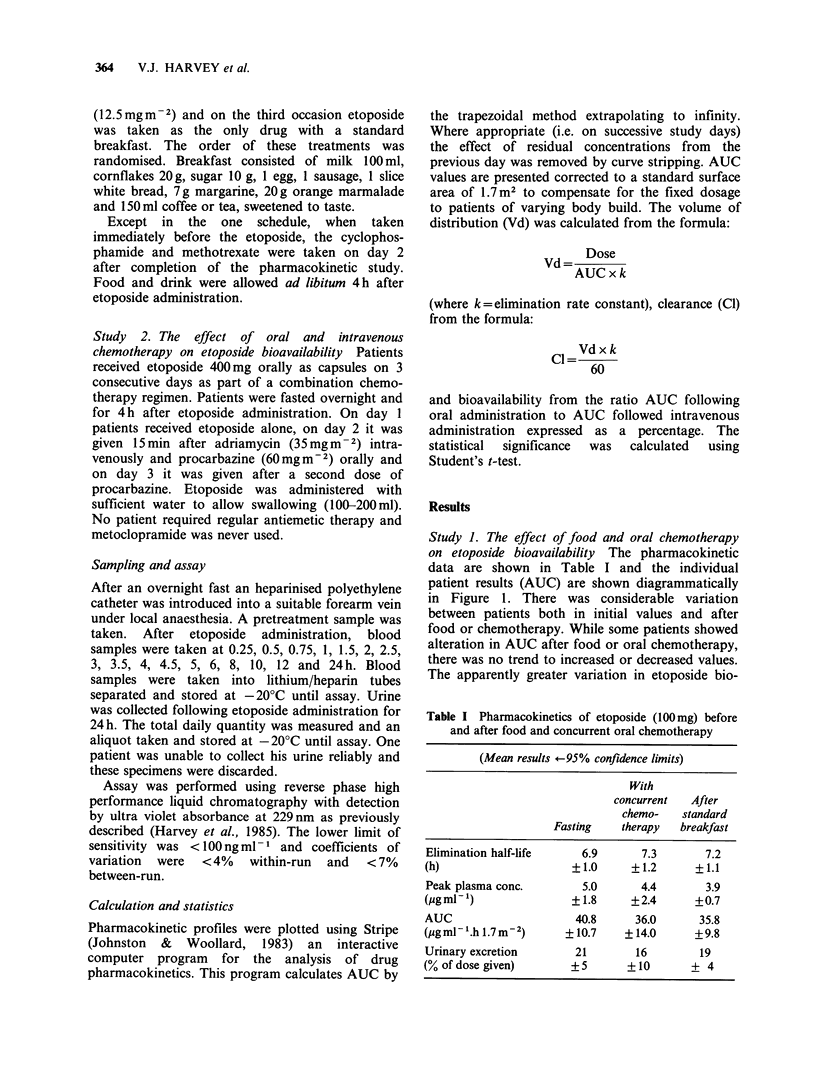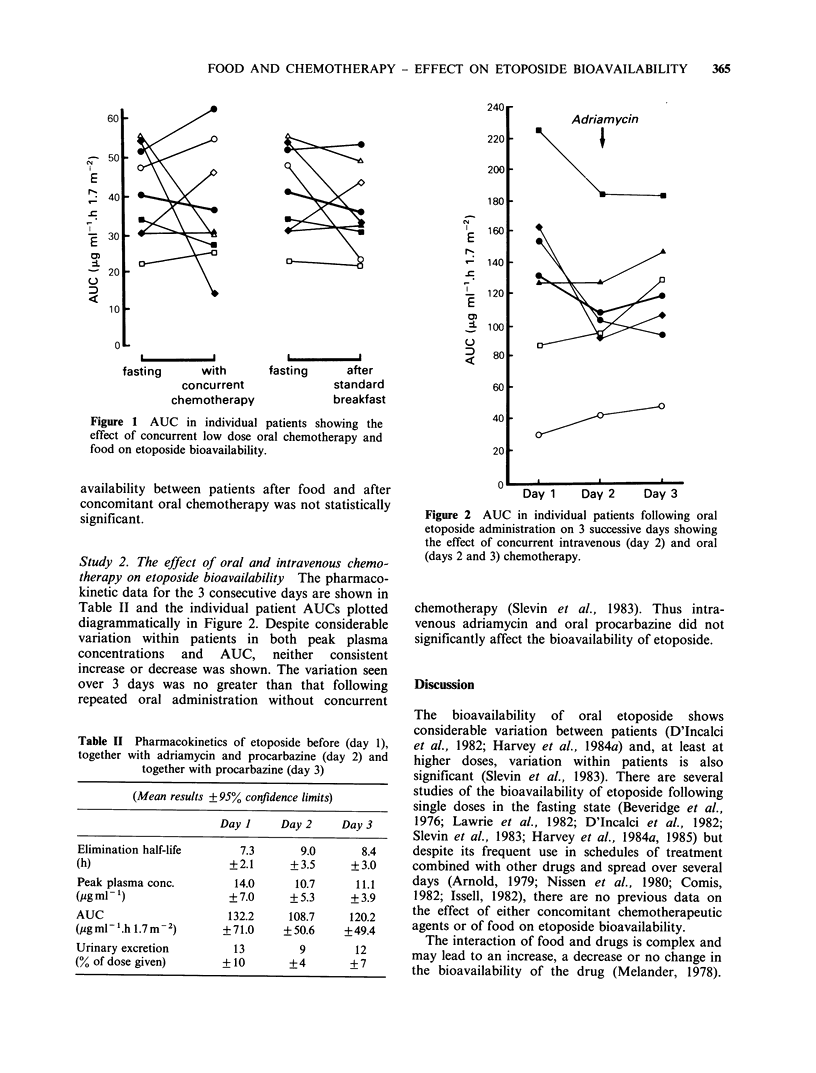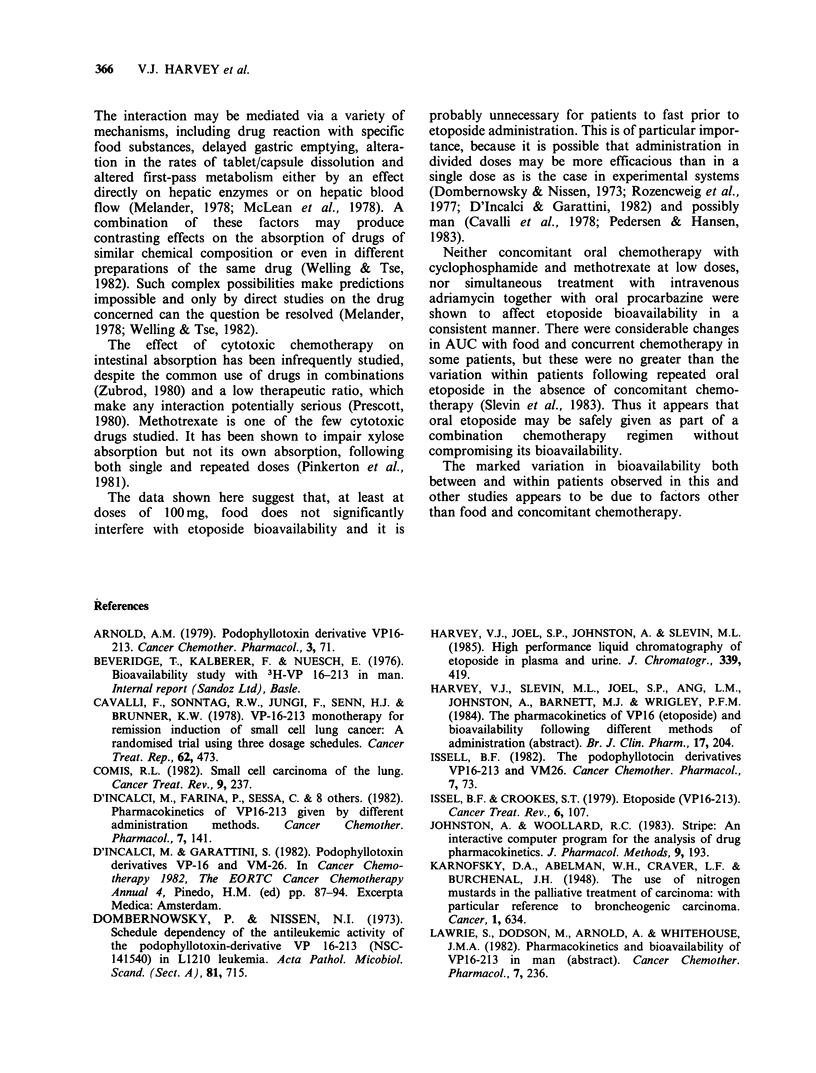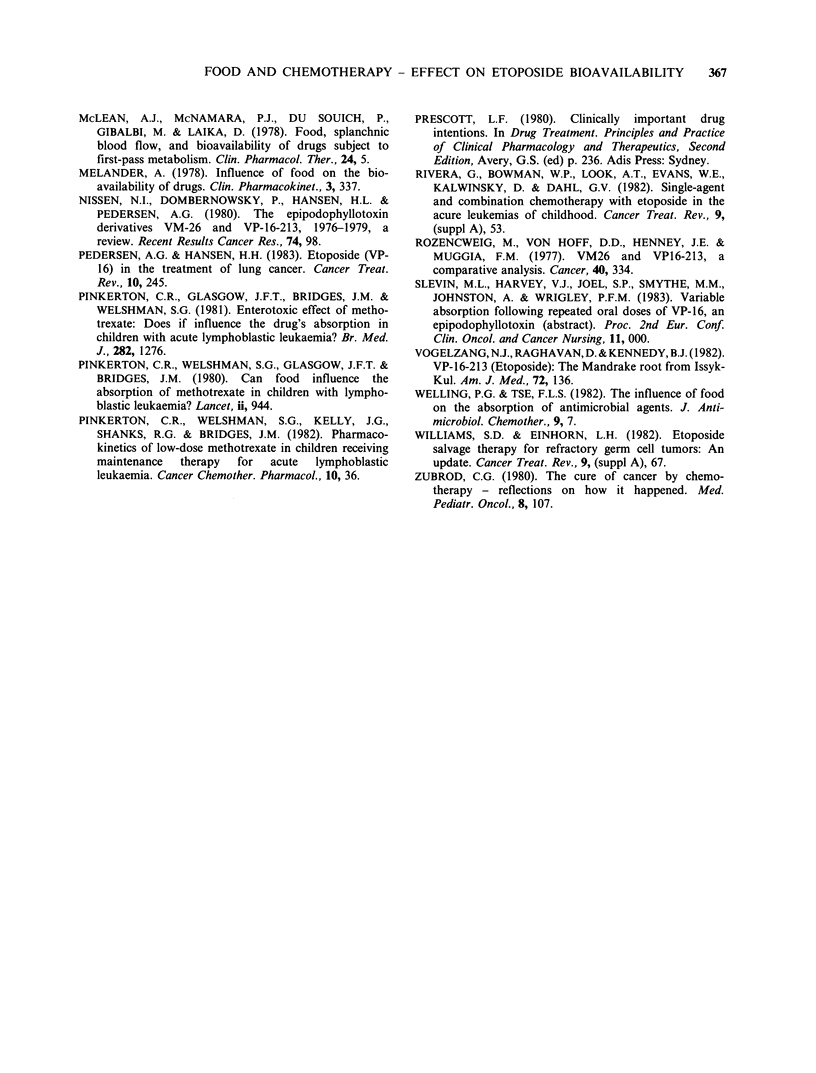# The effect of food and concurrent chemotherapy on the bioavailability of oral etoposide.

**DOI:** 10.1038/bjc.1985.202

**Published:** 1985-09

**Authors:** V. J. Harvey, M. L. Slevin, S. P. Joel, A. Johnston, P. F. Wrigley

## Abstract

There is no information on the effect of food or concurrent drug administration on the bioavailability of oral etoposide, despite the fact that treatment is frequently administered over several days and most often in combination with other cytotoxic agents. The influence of these factors has been studied in 11 patients, receiving combination cytotoxic therapy for extensive small cell lung carcinoma. Neither food nor concurrent oral or intravenous chemotherapy had a significant effect on the mean plasma concentrations of etoposide, achieved following oral administration. Wide variation in peak plasma concentrations and in area under the concentration time curve (AUC) occurred both between and within patients. It appears unnecessary for patients receiving etoposide (at 100 mg) to fast prior to drug administration. Furthermore, oral etoposide (at 100 mg and at 400 mg) may be given in combination with other cytotoxic agents without compromising its bioavailability.


*Br. J. Cancer* (1985), **52**, 363–367

### The effect of food and concurrent chemotherapy on the bioavailability of oral etoposide

V.J. Harvey[1], M.L. Slevin[1], S.P. Joel[1], A. Johnston[2] & P.F.M. Wrigley[1]

[1]*Imperial Cancer Research Fund Department of Medical Oncology, St Bartholomew's and Hackney Hospitals; and* [2]*Department of Clinical Pharmacology, St Bartholomew's Hospital, London EC1A, UK.*

**Summary** There is no information on the effect of food or concurrent drug administration on the bioavailability of oral etoposide, despite the fact that treatment is frequently administered over several days and most often in combination with other cytotoxic agents. The influence of these factors has been studied in 11 patients, receiving combination cytotoxic therapy for extensive small cell lung carcinoma. Neither food nor concurrent oral or intravenous chemotherapy had a significant effect on the mean plasma concentrations of etoposide, achieved following oral administration. Wide variation in peak plasma concentrations and in area under the concentration*time curve (AUC) occurred both between and within patients. It appears unnecessary for patients receiving etoposide (at 100 mg) to fast prior to drug administration. Furthermore, oral etoposide (at 100 mg and at 400 mg) may be given in combination with other cytotoxic agents without compromising its bioavailability.

Etoposide was introduced into clinical trials in the early 1970s (Issell, 1982) and is established in the treatment of several malignancies, including small cell lung cancer, germ cell tumours and lymphomas (Arnold, 1979; Issell & Crooke, 1979; Vogelzang *et al.*, 1982).

The demonstration of schedule dependency in both experimental systems (Dombernowsky & Nissen, 1973; Rozencweig *et al.*, 1977; D'Incalci & Garattini, 1982) and possibly also in man (Cavalli *et al.*, 1978; Pedersen & Hansen, 1983) has led to most schedules of therapy being given over several (usually 3–5) days (Arnold, 1979; Nissen *et al.*, 1980; Issell, 1982).

The bioavailability of the oral etoposide capsule has been shown to be approximately 50% but with large variation between patients (D'Incalci *et al.*, 1982; Harvey *et al.*, 1984*a*). Despite the widespread use of oral etoposide over 3–5 days and its predominant use as part of combination chemotherapy regimens (Arnold, 1979; Comis, 1982; Rivera *et al.*, 1982; Williams & Einhorn, 1982), there are no data concerning the influence of food or other chemotherapy on etoposide bioavailability. The intestinal absorption of some drugs has been shown to be affected by both food (Melander, 1978; McLean *et al.*, 1978; Pinkerton *et al.*, 1980) and chemotherapy (Pinkerton *et al.*, 1982). The effect of food and concomitant oral and intravenous chemotherapy on the bioavailability of etoposide has therefore been studied.

Correspondence: M.L. Slevin
Received 3 April 1985.

### Materials and methods

### *Patients*

Eleven patients receiving chemotherapy for extensive small cell lung carcinoma were studied. All were ambulant (performance score >60% Karnofsky *et al.*, 1948) with normal bone marrow, hepatic and renal function. No patients had disturbance of the gastrointestinal tract. Eight patients receiving primary chemotherapy were studied on 3 separate occasions to assess the effect of food and concomitant oral chemotherapy on etoposide bioavailability (Study 1). Six patients (3 of whom had previously been part of the above study) were receiving therapy for relapsed extensive SCLC and were studied on 3 successive days to assess the effect of intravenous and oral chemotherapy on etoposide bioavailability (Study 2).

### *Treatment*

*Study 1. The effect of food and oral chemotherapy on etoposide bioavailability* Patients received etoposide weekly as part of a combination chemotherapy regimen. Etoposide pharmacokinetics were studied on 3 separate occasions at least one week apart. Each patient thus acted as his own control. Etoposide was administered as a single 100 mg capsule with sufficient water (~50 ml) to allow swallowing. Patients were fasted overnight prior to administration of etoposide. On one occasion etoposide was taken alone following the fast, on another occasion immediately after oral cyclophosphamide (100 mg m$^{-2}$) and oral methotrexate

© The Macmillan Press Ltd., 1985

(12.5 mg m$^{-2}$) and on the third occasion etoposide was taken as the only drug with a standard breakfast. The order of these treatments was randomised. Breakfast consisted of milk 100 ml, cornflakes 20 g, sugar 10 g, 1 egg, 1 sausage, 1 slice white bread, 7 g margarine, 20 g orange marmalade and 150 ml coffee or tea, sweetened to taste.

Except in the one schedule, when taken immediately before the etoposide, the cyclophosphamide and methotrexate were taken on day 2 after completion of the pharmacokinetic study. Food and drink were allowed *ad libitum* 4 h after etoposide administration.

*Study 2. The effect of oral and intravenous chemotherapy on etoposide bioavailability* Patients received etoposide 400 mg orally as capsules on 3 consecutive days as part of a combination chemotherapy regimen. Patients were fasted overnight and for 4 h after etoposide administration. On day 1 patients received etoposide alone, on day 2 it was given 15 min after adriamycin (35 mg m$^{-2}$) intravenously and procarbazine (60 mg m$^{-2}$) orally and on day 3 it was given after a second dose of procarbazine. Etoposide was administered with sufficient water to allow swallowing (100–200 ml). No patient required regular antiemetic therapy and metoclopramide was never used.

### *Sampling and assay*

After an overnight fast an heparinised polyethylene catheter was introduced into a suitable forearm vein under local anaesthesia. A pretreatment sample was taken. After etoposide administration, blood samples were taken at 0.25, 0.5, 0.75, 1, 1.5, 2, 2.5, 3, 3.5, 4, 4.5, 5, 6, 8, 10, 12 and 24 h. Blood samples were taken into lithium/heparin tubes separated and stored at −20°C until assay. Urine was collected following etoposide administration for 24 h. The total daily quantity was measured and an aliquot taken and stored at −20°C until assay. One patient was unable to collect his urine reliably and these specimens were discarded.

Assay was performed using reverse phase high performance liquid chromatography with detection by ultra violet absorbance at 229 nm as previously described (Harvey *et al.*, 1985). The lower limit of sensitivity was <100 ng ml$^{-1}$ and coefficients of variation were <4% within-run and <7% between-run.

### *Calculation and statistics*

Pharmacokinetic profiles were plotted using Stripe (Johnston & Woollard, 1983) an interactive computer program for the analysis of drug pharmacokinetics. This program calculates AUC by the trapezoidal method extrapolating to infinity. Where appropriate (i.e. on successive study days) the effect of residual concentrations from the previous day was removed by curve stripping. AUC values are presented corrected to a standard surface area of 1.7 m$^2$ to compensate for the fixed dosage to patients of varying body build. The volume of distribution (Vd) was calculated from the formula:

$$\mathrm{Vd} = \frac{\mathrm{Dose}}{\mathrm{AUC} \times k}$$

(where $k$ = elimination rate constant), clearance (Cl) from the formula:

$$\mathrm{Cl} = \frac{\mathrm{Vd} \times k}{60}$$

and bioavailability from the ratio AUC following oral administration to AUC followed intravenous administration expressed as a percentage. The statistical significance was calculated using Student's $t$-test.

### Results

*Study 1. The effect of food and oral chemotherapy on etoposide bioavailability* The pharmacokinetic data are shown in Table I and the individual patient results (AUC) are shown diagrammatically in Figure 1. There was considerable variation between patients both in initial values and after food or chemotherapy. While some patients showed alteration in AUC after food or oral chemotherapy, there was no trend to increased or decreased values. The apparently greater variation in etoposide bio-

**Table I** Pharmacokinetics of etoposide (100 mg) before and after food and concurrent oral chemotherapy

(*Mean results* ←*95% confidence limits*)

| | *Fasting* | *With concurrent chemo-therapy* | *After standard breakfast* |
|---|---|---|---|
| Elimination half-life (h) | 6.9 ±1.0 | 7.3 ±1.2 | 7.2 ±1.1 |
| Peak plasma conc. (μg ml$^{-1}$) | 5.0 ±1.8 | 4.4 ±2.4 | 3.9 ±0.7 |
| AUC (μg ml$^{-1}$.h 1.7 m$^{-2}$) | 40.8 ±10.7 | 36.0 ±14.0 | 35.8 ±9.8 |
| Urinary excretion (% of dose given) | 21 ±5 | 16 ±10 | 19 ± 4 |

**Figure 1** AUC in individual patients showing the effect of concurrent low dose oral chemotherapy and food on etoposide bioavailability.

availability between patients after food and after concomitant oral chemotherapy was not statistically significant.

*Study 2. The effect of oral and intravenous chemotherapy on etoposide bioavailability* The pharmacokinetic data for the 3 consecutive days are shown in Table II and the individual patient AUCs plotted diagrammatically in Figure 2. Despite considerable variation within patients in both peak plasma concentrations and AUC, neither consistent increase or decrease was shown. The variation seen over 3 days was no greater than that following repeated oral administration without concurrent chemotherapy (Slevin *et al.*, 1983). Thus intravenous adriamycin and oral procarbazine did not significantly affect the bioavailability of etoposide.

**Table II** Pharmacokinetics of etoposide before (day 1), together with adriamycin and procarbazine (day 2) and together with procarbazine (day 3)

(*Mean results* ±*95% confidence limits*)

| | *Day 1* | *Day 2* | *Day 3* |
|---|---|---|---|
| Elimination half-life (h) | 7.3 ±2.1 | 9.0 ±3.5 | 8.4 ±3.0 |
| Peak plasma conc. (μg ml$^{-1}$) | 14.0 ±7.0 | 10.7 ±5.3 | 11.1 ±3.9 |
| AUC (μg ml$^{-1}$.h 1.7 m$^{-2}$) | 132.2 ±71.0 | 108.7 ±50.6 | 120.2 ±49.4 |
| Urinary excretion (% of dose given) | 13 ±10 | 9 ±4 | 12 ±7 |

**Figure 2** AUC in individual patients following oral etoposide administration on 3 successive days showing the effect of concurrent intravenous (day 2) and oral (days 2 and 3) chemotherapy.

### Discussion

The bioavailability of oral etoposide shows considerable variation between patients (D'Incalci *et al.*, 1982; Harvey *et al.*, 1984*a*) and, at least at higher doses, variation within patients is also significant (Slevin *et al.*, 1983). There are several studies of the bioavailability of etoposide following single doses in the fasting state (Beveridge *et al.*, 1976; Lawrie *et al.*, 1982; D'Incalci *et al.*, 1982; Slevin *et al.*, 1983; Harvey *et al.*, 1984*a*, 1985) but despite its frequent use in schedules of treatment combined with other drugs and spread over several days (Arnold, 1979; Nissen *et al.*, 1980; Comis, 1982; Issell, 1982), there are no previous data on the effect of either concomitant chemotherapeutic agents or of food on etoposide bioavailability.

The interaction of food and drugs is complex and may lead to an increase, a decrease or no change in the bioavailability of the drug (Melander, 1978).

The interaction may be mediated via a variety of mechanisms, including drug reaction with specific food substances, delayed gastric emptying, alteration in the rates of tablet/capsule dissolution and altered first-pass metabolism either by an effect directly on hepatic enzymes or on hepatic blood flow (Melander, 1978; McLean *et al.*, 1978). A combination of these factors may produce contrasting effects on the absorption of drugs of similar chemical composition or even in different preparations of the same drug (Welling & Tse, 1982). Such complex possibilities make predictions impossible and only by direct studies on the drug concerned can the question be resolved (Melander, 1978; Welling & Tse, 1982).

The effect of cytotoxic chemotherapy on intestinal absorption has been infrequently studied, despite the common use of drugs in combinations (Zubrod, 1980) and a low therapeutic ratio, which make any interaction potentially serious (Prescott, 1980). Methotrexate is one of the few cytotoxic drugs studied. It has been shown to impair xylose absorption but not its own absorption, following both single and repeated doses (Pinkerton *et al.*, 1981).

The data shown here suggest that, at least at doses of 100 mg, food does not significantly interfere with etoposide bioavailability and it is probably unnecessary for patients to fast prior to etoposide administration. This is of particular importance, because it is possible that administration in divided doses may be more efficacious than in a single dose as is the case in experimental systems (Dombernowsky & Nissen, 1973; Rozencweig *et al.*, 1977; D'Incalci & Garattini, 1982) and possibly man (Cavalli *et al.*, 1978; Pedersen & Hansen, 1983).

Neither concomitant oral chemotherapy with cyclophosphamide and methotrexate at low doses, nor simultaneous treatment with intravenous adriamycin together with oral procarbazine were shown to affect etoposide bioavailability in a consistent manner. There were considerable changes in AUC with food and concurrent chemotherapy in some patients, but these were no greater than the variation within patients following repeated oral etoposide in the absence of concomitant chemotherapy (Slevin *et al.*, 1983). Thus it appears that oral etoposide may be safely given as part of a combination chemotherapy regimen without compromising its bioavailability.

The marked variation in bioavailability both between and within patients observed in this and other studies appears to be due to factors other than food and concomitant chemotherapy.

### References

ARNOLD, A.M. (1979). Podophyllotoxin derivative VP16-213. *Cancer Chemother. Pharmacol.*, **3**, 71.

BEVERIDGE, T., KALBERER, F. & NUESCH, E. (1976). Bioavailability study with $^{3}$H-VP 16–213 in man. *Internal report (Sandoz Ltd), Basle.*

CAVALLI, F., SONNTAG, R.W., JUNGI, F., SENN, H.J. & BRUNNER, K.W. (1978). VP-16-213 monotherapy for remission induction of small cell lung cancer: A randomised trial using three dosage schedules. *Cancer Treat. Rep.*, **62**, 473.

COMIS, R.L. (1982). Small cell carcinoma of the lung. *Cancer Treat. Rev.*, **9**, 237.

D'INCALCI, M., FARINA, P., SESSA, C. & 8 others. (1982). Pharmacokinetics of VP16-213 given by different administration methods. *Cancer Chemother. Pharmacol.*, **7**, 141.

D'INCALCI, M. & GARATTINI, S. (1982). Podophyllotoxin derivatives VP-16 and VM-26. In *Cancer Chemotherapy 1982, The EORTC Cancer Chemotherapy Annual 4*, Pinedo, H.M. (ed) pp. 87–94. Excerpta Medica: Amsterdam.

DOMBERNOWSKY, P. & NISSEN, N.I. (1973). Schedule dependency of the antileukemic activity of the podophyllotoxin-derivative VP 16-213 (NSC-141540) in L1210 leukemia. *Acta Pathol. Micobiol. Scand. (Sect. A)*, **81**, 715.

HARVEY, V.J., JOEL, S.P., JOHNSTON, A. & SLEVIN, M.L. (1985). High performance liquid chromatography of etoposide in plasma and urine. *J. Chromatogr.*, **339**, 419.

HARVEY, V.J., SLEVIN, M.L., JOEL, S.P., ANG, L.M., JOHNSTON, A., BARNETT, M.J. & WRIGLEY, P.F.M. (1984). The pharmacokinetics of VP16 (etoposide) and bioavailability following different methods of administration (abstract). *Br. J. Clin. Pharm.*, **17**, 204.

ISSELL, B.F. (1982). The podophyllotocin derivatives VP16-213 and VM26. *Cancer Chemother. Pharmacol.*, **7**, 73.

ISSEL, B.F. & CROOKES, S.T. (1979). Etoposide (VP16-213). *Cancer Treat. Rev.*, **6**, 107.

JOHNSTON, A. & WOOLLARD, R.C. (1983). Stripe: An interactive computer program for the analysis of drug pharmacokinetics. *J. Pharmacol. Methods*, **9**, 193.

KARNOFSKY, D.A., ABELMAN, W.H., CRAVER, L.F. & BURCHENAL, J.H. (1948). The use of nitrogen mustards in the palliative treatment of carcinoma: with particular reference to broncheogenic carcinoma. *Cancer*, **1**, 634.

LAWRIE, S., DODSON, M., ARNOLD, A. & WHITEHOUSE, J.M.A. (1982). Pharmacokinetics and bioavailability of VP16-213 in man (abstract). *Cancer Chemother. Pharmacol.*, **7**, 236.

McLEAN, A.J., McNAMARA, P.J., DU SOUICH, P., GIBALBI, M. & LAIKA, D. (1978). Food, splanchnic blood flow, and bioavailability of drugs subject to first-pass metabolism. *Clin. Pharmacol. Ther.*, **24**, 5.

MELANDER, A. (1978). Influence of food on the bioavailability of drugs. *Clin. Pharmacokinet.*, **3**, 337.

NISSEN, N.I., DOMBERNOWSKY, P., HANSEN, H.L. & PEDERSEN, A.G. (1980). The epipodophyllotoxin derivatives VM-26 and VP-16-213, 1976–1979, a review. *Recent Results Cancer Res.*, **74**, 98.

PEDERSEN, A.G. & HANSEN, H.H. (1983). Etoposide (VP-16) in the treatment of lung cancer. *Cancer Treat. Rev.*, **10**, 245.

PINKERTON, C.R., GLASGOW, J.F.T., BRIDGES, J.M. & WELSHMAN, S.G. (1981). Enterotoxic effect of methotrexate: Does if influence the drug's absorption in children with acute lymphoblastic leukaemia? *Br. Med. J.*, **282**, 1276.

PINKERTON, C.R., WELSHMAN, S.G., GLASGOW, J.F.T. & BRIDGES, J.M. (1980). Can food influence the absorption of methotrexate in children with lymphoblastic leukaemia? *Lancet*, **ii**, 944.

PINKERTON, C.R., WELSHMAN, S.G., KELLY, J.G., SHANKS, R.G. & BRIDGES, J.M. (1982). Pharmacokinetics of low-dose methotrexate in children receiving maintenance therapy for acute lymphoblastic leukaemia. *Cancer Chemother. Pharmacol.*, **10**, 36.

PRESCOTT, L.F. (1980). Clinically important drug intentions. In *Drug Treatment. Principles and Practice of Clinical Pharmacology and Therapeutics, Second Edition*, Avery, G.S. (ed) p. 236. Adis Press: Sydney.

RIVERA, G., BOWMAN, W.P., LOOK, A.T., EVANS, W.E., KALWINSKY, D. & DAHL, G.V. (1982). Single-agent and combination chemotherapy with etoposide in the acure leukemias of childhood. *Cancer Treat. Rev.*, **9**, (suppl A), 53.

ROZENCWEIG, M., VON HOFF, D.D., HENNEY, J.E. & MUGGIA, F.M. (1977). VM26 and VP16-213, a comparative analysis. *Cancer*, **40**, 334.

SLEVIN, M.L., HARVEY, V.J., JOEL, S.P., SMYTHE, M.M., JOHNSTON, A. & WRIGLEY, P.F.M. (1983). Variable absorption following repeated oral doses of VP-16, an epipodophyllotoxin (abstract). *Proc. 2nd Eur. Conf. Clin. Oncol. and Cancer Nursing*, **11**, 000.

VOGELZANG, N.J., RAGHAVAN, D. & KENNEDY, B.J. (1982). VP-16-213 (Etoposide): The Mandrake root from Issyk-Kul. *Am. J. Med.*, **72**, 136.

WELLING, P.G. & TSE, F.L.S. (1982). The influence of food on the absorption of antimicrobial agents. *J. Antimicrobiol. Chemother.*, **9**, 7.

WILLIAMS, S.D. & EINHORN, L.H. (1982). Etoposide salvage therapy for refractory germ cell tumors: An update. *Cancer Treat. Rev.*, **9**, (suppl A), 67.

ZUBROD, C.G. (1980). The cure of cancer by chemotherapy – reflections on how it happened. *Med. Pediatr. Oncol.*, **8**, 107.